# Focal adhesion kinase confers pro‐migratory and antiapoptotic properties and is a potential therapeutic target in Ewing sarcoma

**DOI:** 10.1002/1878-0261.12610

**Published:** 2019-12-21

**Authors:** Konrad Steinestel, Marcel Trautmann, Esther‐Pia Jansen, Uta Dirksen, Jan Rehkämper, Jan‐Henrik Mikesch, Julia S. Gerke, Martin F. Orth, Giuseppina Sannino, Maria‐Francisca Arteaga, Claudia Rossig, Eva Wardelmann, Thomas G. P. Grünewald, Wolfgang Hartmann

**Affiliations:** ^1^ Gerhard Domagk Institute of Pathology University Hospital Münster Germany; ^2^ Institute of Pathology and Molecular Pathology Bundeswehrkrankenhaus Ulm Germany; ^3^ Division of Translational Pathology Gerhard‐Domagk‐Institute of Pathology University Hospital Münster Germany; ^4^ Pediatrics III West German Cancer Centre University Hospital Essen Germany; ^5^ Institute of Pathology University Hospital Cologne Germany; ^6^ Department of Medicine A University Hospital Münster Germany; ^7^ Max Eder Research Group for Pediatric Sarcoma Biology Institute of Pathology Faculty of Medicine LMU Munich Germany; ^8^ Department of Pediatric Hematology and Oncology University Children's Hospital Münster Germany; ^9^ German Cancer Consortium (DKTK), Partner Site Munich Germany; ^10^ German Cancer Research Center (DKFZ) Heidelberg Germany; ^11^ Institute of Pathology Faculty of Medicine LMU Munich Germany

**Keywords:** cytoskeleton, Ewing sarcoma, focal adhesion kinase, metastasis, migration

## Abstract

Oncogenesis of Ewing sarcoma (EwS), the second most common malignant bone tumor of childhood and adolescence, is dependent on the expression of chimeric EWSR1‐ETS fusion oncogenes, most often EWSR1‐FLI1 (E/F). E/F expression leads to dysregulation of focal adhesions (FAs) enhancing the migratory capacity of EwS cells. Here, we show that, in EwS cell lines and tissue samples, focal adhesion kinase (FAK) is expressed and phosphorylated at Y397 in an E/F‐dependent way involving Ezrin. Employing different EwS cell lines as *in vitro* models, we found that key malignant properties of E/F are mediated via substrate‐independent autophosphorylation of FAK on Y397. This phosphorylation results in enhanced FA formation, Rho‐dependent cell migration, and impaired caspase‐3‐mediated apoptosis *in vitro*. Conversely, treatment with the FAK inhibitor 15 (1,2,4,5‐benzenetetraamine tetrahydrochloride (Y15) enhanced caspase‐mediated apoptosis and EwS cell migration, independent from the respective EWSR1‐ETS fusion type, mimicking an anoikis‐like phenotype and paralleling the effects of FAK siRNA knockdown. Our findings were confirmed *in vivo* using an avian chorioallantoic membrane model and provide a first rationale for the therapeutic use of FAK inhibitors to impair metastatic dissemination of EwS.

## Graphical abstract

In Ewing sarcoma (EwS) cells, EWSR1‐FLI1‐dependent expression of Ezrin contributes to autophosphorylation of focal adhesion kinase (FAK) on tyrosine 397. This impairs apoptosis and detachment‐dependent cell death (anoikis) and enhances focal adhesion formation and migratory capacity of EwS. Since Y397 autophosphorylation can effectively be targeted by FAK inhibitor 15 (1,2,4,5‐benzenetetraamine tetrahydrochloride (Y15), FAK might represent a valuable target for antimetastatic pharmacotherapy in EwS.

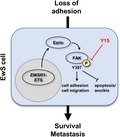

**Abbreviations**
CAM: chorioallantoic membrane
E/F: EWSR1‐FLI1 fusion oncogene
EwS: Ewing sarcoma
FACS: fluorescence‐activated cell sorting
FAK: focal adhesion kinase
TMA: tissue microarray
Y15: FAK inhibitor 15 (1,2,4,5‐benzenetetraamine tetrahydrochloride

## 1 Introduction

Ewing sarcoma (EwS) is a highly aggressive cancer of bone and soft tissue that predominantly affects children and adolescents. Frequently, (micro)metastasis is already present at the time of diagnosis, and no effective therapy strategies have been established for these patients yet (Dahlin *et al.*, 1961; Wang and Schulz, 1953). Knowledge of the biological mechanisms underlying EwS cell migration might provide rationales for developing urgently needed targeted therapies to prevent or to slow down metastatic dissemination of EwS cells.

Ewing sarcoma are genetically stable tumors characterized by a chromosomal translocation leading to fusion of the *EWSR1* gene on chromosome 22 and variable members of the ETS family of transcription factors. In 85% of cases, the translocation partner for *EWSR1* is *FLI1* on chromosome 11; other possible fusion partners are the *ERG, FEV,* or *ETV1/4* genes (May *et al.*, 1993; Sankar and Lessnick, 2011). The resulting chimeric transcription factor *EWSR1‐FLI1* (E/F) modulates the expression of a large number of target genes, leading to oncogenic transformation of the cell harboring this fusion (Braun *et al.*, 1995).

So far, only a few studies have investigated the effect of E/F expression on cell migration and invasion of EwS cells as a prerequisite to metastasis. Using an RNA interference approach, Chaturvedi *et al.* demonstrated that knockdown of E/F enhanced tumor cell adhesion and spreading; in a subsequent study, the same group showed that E/F‐induced repression of Zyxin and α5 integrin impairs cell adhesion and actin cytoskeletal integrity, thus supporting anchorage‐independent cell growth (Chaturvedi *et al.*, 2014; Chaturvedi *et al.*, 2012). CXCR4‐dependent migration and invasion of EwS cells were shown to depend on the activity of small Rho‐GTPases RAC and CDC42 (Krook *et al.*, 2014).

Focal adhesion kinase (FAK) is a substrate of SRC kinase and localizes to focal adhesions (FAs), where it is activated by integrins, resulting in FA reorganization and increased cell motility (Mitra *et al.*, 2005). Integrin‐mediated autophosphorylation of FAK on Y397 inhibits detachment‐dependent apoptosis, a process named anoikis (Frisch *et al.*, 1996). There is experimental evidence that autophosphorylation of FAK on Y397 results from dimerization of two FAK molecules (Toutant *et al.*, 2002). Alternatively, FAK autophosphorylation can be induced by the FAK interaction partner Ezrin independent from cell‐matrix adhesion (Poullet *et al.*, 2001). Ectopic expression of constitutively active FAK rescues cancer cells from induced anoikis (Frisch *et al.*, 1996). Moreover, FAK activation appears to be essential for migration as well as mechano‐induced osteogenic differentiation of mesenchymal stem cells that are believed to constitute the cells of origin in EwS (Riggi *et al.*, 2008; Shih *et al.*, 2011; Wang *et al.*, 2015). One report previously identified FAK as a potential therapeutic target in EwS by the use of high‐throughput tyrosine kinase activity profiling (Crompton *et al.*, 2013). The same group just recently showed that synergistic inhibition of FAK and Aurora B kinase effectively impairs EwS growth in multiple xenograft models, while another study revealed that dual inhibition of FAK and insulin‐like growth factor‐I receptor acts synergistically with conventional chemotherapy to induce apoptosis and impair invasion of EwS *in vitro* and *in vivo* (Moritake *et al.*, 2019; Wang *et al.*, 2019). In line, another report showed that miR‐138, via targeting *FAK*, inhibits proliferation and mobility and induces anoikis of EwS cells (Tanaka *et al.*, 2016). However, the exact mechanistic role of FAK in EwS remains elusive.

In this manuscript, we show that E/F‐dependent autophosphorylation of FAK is a crucial mechanism underlying EwS aggressivity as it promotes a migratory phenotype and inhibits caspase‐mediated apoptosis. We show that this mechanism can effectively be targeted by the FAK inhibitor 15 (1,2,4,5‐benzenetetraamine tetrahydrochloride (Y15), independent from the respective *EWSR1‐ETS* fusion type. Hence, our results point toward a possible use of FAK inhibitors to prevent metastatic dissemination in EwS.

## 2 Materials and methods

### 2.1 DOX‐inducible knockdown and *in vivo* xenografts

A673/TR/shEF1 cells (Carrillo *et al.*, 2007), which contain a doxycycline (DOX)‐inducible shRNA against *EWSR1‐FLI1*, were injected subcutaneously in the flanks of immunocompromised NSG (NOD/scid/gamma) mice. When tumors reached an average volume of 180 mm^3^, mice were randomized and either received 2 mg·mL^−1^ DOX (Sigma, Darmstadt, Germany) and 5% sucrose in the drinking water (DOX +) or only 5% sucrose (DOX −). Mice were sacrificed after 96 h, and tumors were isolated for RNA and histological analysis. RNA was extracted using the ReliaPrep miRNA Cell and Tissue Miniprep System (Promega, Mannheim, Germany). Knockdown of *EWSR1‐FLI1* was confirmed by qRT–PCR (Grünewald *et al.*, 2015). The transcriptome of each tumor (*n* = 3 for DOX+ and DOX–) was profiled on Affymetrix Clariom D arrays (RIN > 9). Microarray data were normalized on gene level using Signal Space Transformation Robust Multi‐Chip Average and Affymetrix CDF. Three FFPE cores (1 mm) were taken from each xenograft tumor to create a tissue microarray (TMA). Animal experiments were conducted in accordance with the recommendations of the European Community (86/609/EEC), the Government of Upper Bavaria (Germany), and UKCCCR (guidelines for the welfare and use of animals in cancer research).

### 2.2 Immunohistochemistry of EwS patient samples

Immunohistochemistry was performed on TMA slides containing at least two representative cores (1 mm) derived from a FFPE tissue samples from 97 EwS patients from the cooperative Ewing sarcoma study (CESS) group with full clinical and follow‐up information; clinicopathological data are given in Table S1. This study was approved by the Ethical Committee of the University of Münster (No. 2014‐668‐f‐S) and conducted in accordance with current ethical standards (Declaration of Helsinki, 1975) and with the understanding and written consent of each subject.

Tissue microarray were stained using anti‐FAK/phospho‐FAK/Ezrin antibodies (1 : 250; Cell Signaling, Frankfurt, Germany). Lung adenocarcinoma tissue was used as positive control. Immunohistochemical (IHC) staining was graded as strong (2; intense staining in ≥ 50% of tumor cells), moderate (1; intermediate to intense staining in 1–49% of tumor cells), and negative (0, no staining or staining in < 1% of tumor cells). Software‐based quantification was performed with the reciprocal intensity method (imagej; NIH, Bethesda, MD, USA) (Nguyen *et al.*, 2013).

### 2.3 Cell lines and cell‐based analyses

A673, TC‐32 (*EWSR1‐FLI1* translocated), and CADO‐ES1 (*EWSR1‐ERG* translocated) cells have been described before (Kodama *et al.*, 1994; Matsui *et al.*, 2003; May *et al.*, 1993; Ren *et al.*, 2016). Cells were grown in DMEM with 10% FBS and RPMI medium with 15% FBS, respectively. All cell lines were authenticated using STR analysis (data not shown). For cultivation under adhesion‐free conditions, we used Softwell AF adhesion‐free hydrogel plates (#SW6‐AF; Matrigen Technologies, Brea, CA, USA).

#### 2.3.1 Western immunoblotting and PathScan Array

Western blotting was performed using a routine protocol after cell lysis in RIPA buffer including protease and phosphatase inhibitors (#9806 and #5872; Cell Signaling). The PathScan® RTK Signaling Antibody Array Kit (Chemiluminescent Readout, #7982; Cell Signaling) was used with the identical cell lysates for simultaneous detection of phosphorylated and cleaved signaling molecules following the manufacturer’s protocol and readout with a chemiluminescence detection system (Bio‐Rad, Munich, Germany). Grayscale intensity values were normalized to internal positive controls and measured using imagej software (NIH). Table S2 summarizes the antibodies and dilutions that were used in the study.

#### 2.3.2 Protein co‐immunoprecipitation

Co‐immunoprecipitation (IP) experiments were performed using the µMACS Protein A/G MicroBeads Kit (Miltenyi Biotec, Bergisch Gladbach, Germany) according to the manufacturer’s instructions as previously described (Stock *et al.*, 2019). Whole‐cell lysate of 5 × 10^6^ cells was mixed with anti‐FAK/anti‐Paxillin rabbit monoclonal antibodies (Cell Signaling; each 1 µg per 500 µg protein lysate) and 50 µL Protein G MicroBeads and incubated for 1 h on ice. For magnetic IP, cell lysate was passed over a separation column placed in the magnetic field of a µMACS separator and washed four times with 200 µL of µMACS cell lysis buffer. The immune complex was then eluted from the column using preheated 1 × SDS sample buffer. Mouse monoclonal antibodies were used for subsequent immunoblotting of interacting proteins as described above to avoid the detection of interfering IgG bands from the IP.

#### 2.3.3 siRNA knockdown

A673, TC‐32, and CADO‐ES1 EwS cells were grown in 25‐cm^2^ cell culture flasks (medium supplemented with 2% FBS) and transfected with indicated siRNA (25 pmol; cell density of 50%) using Lipofectamine RNAiMAX (Life Technologies, Carlsbad, CA, USA). A set of prevalidated short interfering RNAs (Silencer Select siRNA; Life Technologies) was used to knock down EZR (Entrez Gene ID 7430, siRNA IDs s14796 and s14797) and FAK (Entrez Gene ID 5747, siRNA IDs s11485 and s707). Nontargeting control siRNA (BLOCK‐iT Alexa Fluor Red Fluorescent Control; Life Technologies) was included to screen for unspecific off‐target effects. After incubation for 48–72 h, siRNA‐transfected cells were lysed and knockdown efficiency was documented by western immunoblotting as described above.

#### 2.3.4 xCELLigence system

The xCELLigence system (OLS, Bremen, Germany) was used for real‐time, label‐free monitoring of cell health and behavior as previously described (Stock *et al.*, 2019). Cells were seeded on E‐Plate 16 (cell proliferation and adhesion) or into the upper chamber of CIM‐Plate 16 (cell migration). Cell proliferation, morphology change, and attachment quality were measured using electrical impedance; cell migration was monitored using the electronically integrated Boyden chamber of CIM‐Plate 16 with 10% FBS as chemoattractant in the lower chamber. All experiments were performed in quadruplicate using both 20 000 and 40 000 cells for each experimental condition.

#### 2.3.5 Immunofluorescence (IF) microscopy and focal adhesion (FA) quantification and measurement

Immunofluorescence staining was performed as previously described (Liebau *et al.*, 2011). The number of Paxillin‐positive FAs was quantified using imagej (NIH) following a published protocol with slight modifications (Ramadhani *et al.*, 2012): In short, (a) 30 randomly selected cells were photographed, (b) channels were split, (c) outlines of Paxillin‐positive FAs were detected, and (d) objects meeting the predefined criteria for FAs (size, circularity) were counted automatically for each cell. The number of FAs was normalized to the respective cell area. FA diameter was measured using the measurement tool of imagej.

#### 2.3.6 ApoTox‐Glo® assay

The ApoTox‐Glo® assay (Promega) is a multiplex method that simultaneously detects three different protease activities as markers for viable, necrotic, and apoptotic cells in a single sample and was used following the manufacturer’s protocol. A673 EwS cells were treated with Y15 (10 µm) or volume‐adapted concentrations of DMSO for 24 h. Free AFC as a marker of viable cells was determined by the measurement of fluorescence at 400 nm excitation/505 nm emission wavelengths; free R110 as a marker of necrotic cells was measured at 485 nm excitation/520 nm emission. Thirty minutes after adding Caspase‐Glo® 3/7 reagent, the release of aminoluciferin was measured using a luminometer. All tests were performed in triplicate and normalized to control (background), vehicle (DMSO), and Y15 compound controls.

#### 2.3.7 Flow cytometry analyses

The effects of Y15 on the apoptotic rate were assessed by flow cytometric analyses. Annexin V analysis was performed on an Attune NxT Flow Cytometer (Invitrogen, Carlsbad, CA, USA) using the PE Annexin V Apoptosis Detection Kit I (BD Pharmingen, San Jose, CA, USA) following the manufacturer’s instructions. Analysis of cleaved PARP (Asp214; BD Biosciences; phycoerythrin‐labeled) was performed essentially as described before (Trautmann *et al.*, 2017).

#### 2.3.8 Rho activity assay

Rho activity was assessed using the Rho activity assay from Cell Signaling (#8820; Cell Signaling). GTPase activity was measured based on the ability of the GTP‐bound (active) form of Rho to bind Rhotekin‐RBD fusion protein; this was then immunoprecipitated with glutathione resin. The level of Rho as detected in subsequent western immunoblotting correlates with its activation state.

### 2.4 Reagents

Y15 (1,2,4,5‐benzenetetraamine tetrahydrochloride; FAK inhibitor 14, Cat. No. 3414) was obtained in pharmaceutical purity from Tocris Bioscience (Bristol, UK).

### 2.5 Chorioallantoic membrane (CAM) model

Chorioallantoic membrane assays were performed as previously described (Isachenko *et al.*, 2012). Fertilized eggs of White Leghorn chickens were incubated at 37 °C with 60% relative humidity and prepared for implantation on day 4 of incubation. Standard microbiology assessment was performed to exclude subclinical infections. Each egg was washed with warm 70% EtOH, and a hole was drilled through the pointed pole of the shell. The following day, the CAM was exposed by peeling a 1.5‐ to 2.0‐cm window in the shell. This window was covered with tape and the incubation continued. On day 8 of incubation, DMSO/Y15‐treated A673 EwS cells were dissolved in Matrigel and introduced in the CAM. Tumor growth was assessed macroscopically during the following days. After 5 days, tumors were harvested, measured, and weighed, fixed in 5% PFA, and processed for histopathological examination with H/E, FAK/pFAK (Y397), and Ezrin staining as described above. Tumors were measured macroscopically and microscopically using imagej software (NIH).

### 2.6 Statistical analyses

Survival analyses (Kaplan–Meier method) for the patient‐derived sample cohorts were done with spss (IBM, Mannheim, Germany). All statistical analyses for the *in vitro* data were performed using graphpad software (GraphPad, La Jolla, CA, USA). We used chi‐square/Fisher’s exact test for the comparison of categorical variables, while Student’s *t*‐test/Kruskal–Wallis test was performed to compare continuous variables between two groups/more than two groups, respectively. A *P* value < 0.05 was regarded as statistically significant. For the readout of cell viability, toxicity, and apoptosis in the ApoTox‐Glo triplex assay as well as for fluorescence‐activated cell sorting (FACS) analyses, values were calculated after baseline normalization and depicted as ratio of control (100%).

## 3 Results

### 3.1 Doxycycline (DOX)‐inducible downregulation of *EWSR1‐FLI‐1* (E/F) attenuates Ezrin expression and Y397 phosphorylation of FAK in A673 EwS cells

**Figure 1:**
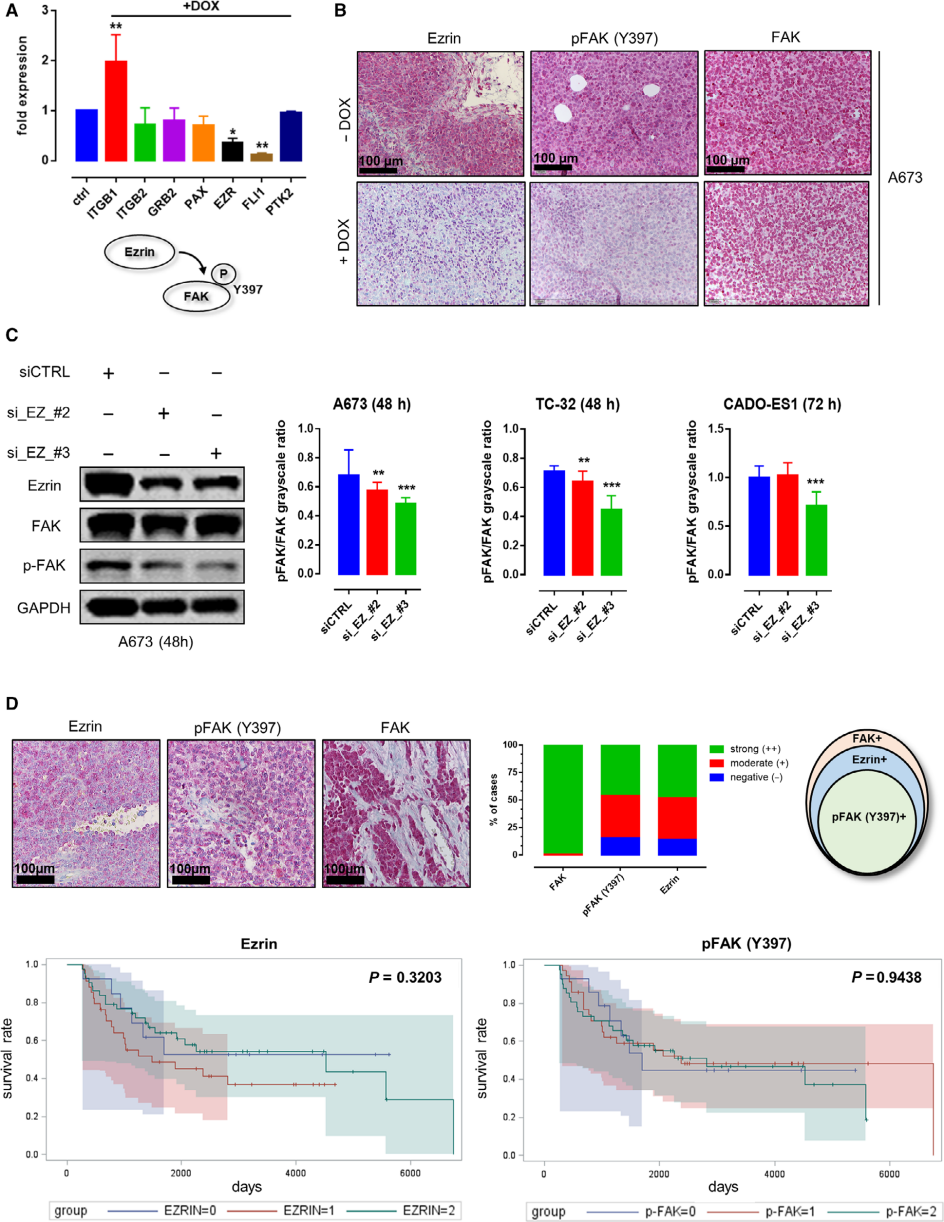
EWSR1‐FLI1‐dependent Ezrin expression and FAK Y397 phosphorylation in an EwS *in vivo* model and primary human tumor samples. (A) DOX‐inducible knockdown of EWSR1‐FLI1 in A673 cells showed downregulation of FLI‐1 and Ezrin mRNA levels in A673 EwS cells, while FAK levels remained unaffected (each *n* = 3, Dunnett’s multiple comparison test). (B) Immunohistochemistry confirmed the downregulation of Ezrin on protein level along with a decrease in Y397 phosphorylation of FAK in A673‐derived xenografts, while FAK protein levels remained unaltered (each *n* = 3, Dunnett’s multiple comparison test). (C) siRNA‐based knockdown of Ezrin led to a significant decrease in Y397 phosphorylation of FAK in EwS cells. (D) IHC analyses of FAK/Ezrin expression and Y397 phosphorylation of FAK in tissue samples derived from 97 EwS patients treated in the CESS trial. All EwS tumor samples were positive for FAK protein in immunohistochemistry, and the majority of tumor samples showed moderate‐to‐strong expression of Ezrin and Y397 phosphorylation of FAK (85% and 84% of cases, respectively). All pFAK+ cases were Ezrin+. Survival analyses showed no significant association between Ezrin expression/FAK phosphorylation and overall survival (*P* = 0.3203 and 0.9438, respectively; *n* = 97; Kaplan–Meier method). *p < 0.05; **p < 0.01; ***p < 0.001.

In order to investigate E/F‐dependent changes in FA gene expression levels, a DOX‐inducible E/F knockdown in A673 EwS cells was performed, revealing decreased levels of *Ezrin* mRNA upon E/F knockdown (Fig. 1A). Correspondingly, IHC analyses of xenograft tumors derived from A673 cells in immunocompromised NSG (NOD/scid/gamma) mice showed significantly decreased Ezrin protein expression and Y397 phosphorylation of FAK upon loss of E/F while total FAK protein expression was unaffected (Fig. 1B).

### 3.2 Y397 phosphorylation of FAK in EwS cells depends on the presence of Ezrin

For further *in vitro* studies, we employed the EwS cell lines A673, TC‐32 (EWSR1‐FLI1 fusion type 1), and CADO‐ES1 (EWSR1‐ERG fusion gene) (See Fig. S1 for comprehensive characterization of all investigated cell lines with respect to proliferative and migratory capacity, FA number, and localization as well as FA protein composition) (Matsui *et al.*, 2003; Smith *et al.*, 2006). All EwS cell lines strongly expressed Ezrin (Fig. S1F). siRNA‐based knockdown of Ezrin significantly impaired Y397 phosphorylation of FAK in A673, TC‐32, and CADO‐ES1 cells (Figs 1C and S1B). Notably, in CADO‐ES1 cells, the effect on Y397 phosphorylation was limited to one Ezrin siRNA (#3) and could only be observed 72 h after transfection.

### 3.3 FAK is ubiquitously expressed and phosphorylated at Y397 in Ezrin‐positive EwS tumor samples

To investigate the relevance of our *in vitro* and *ex vivo* findings in patient samples, IHC analyses were performed in a set of tumors homogeneously treated in the CESS group (*n* = 97; Fig. 1D; see Table S1 for clinicopathological data). All investigated samples showed moderate‐to‐strong FAK staining with Ezrin being expressed in 85% of evaluable tumors. Phosphorylated FAK (Y397) was detected in 84% of samples, which were all simultaneously Ezrin‐positive (*P* < 0.0001, chi‐square test). There was no significant correlation between FAK expression levels, Ezrin expression, or phosphorylation of FAK and overall survival (Fig. 1D) or other clinicopathological characteristics (response to chemotherapy, first relapse, or presence of metastatic disease at the time of diagnosis).

### 3.4 EwS cell lines show adhesion‐independent and fusion type‐independent Y397 phosphorylation of FAK that can be targeted by 1,2,4,5‐benzenetetraamine tetrahydrochloride (FAK inhibitor 14, Y15)

**Figure 2:**
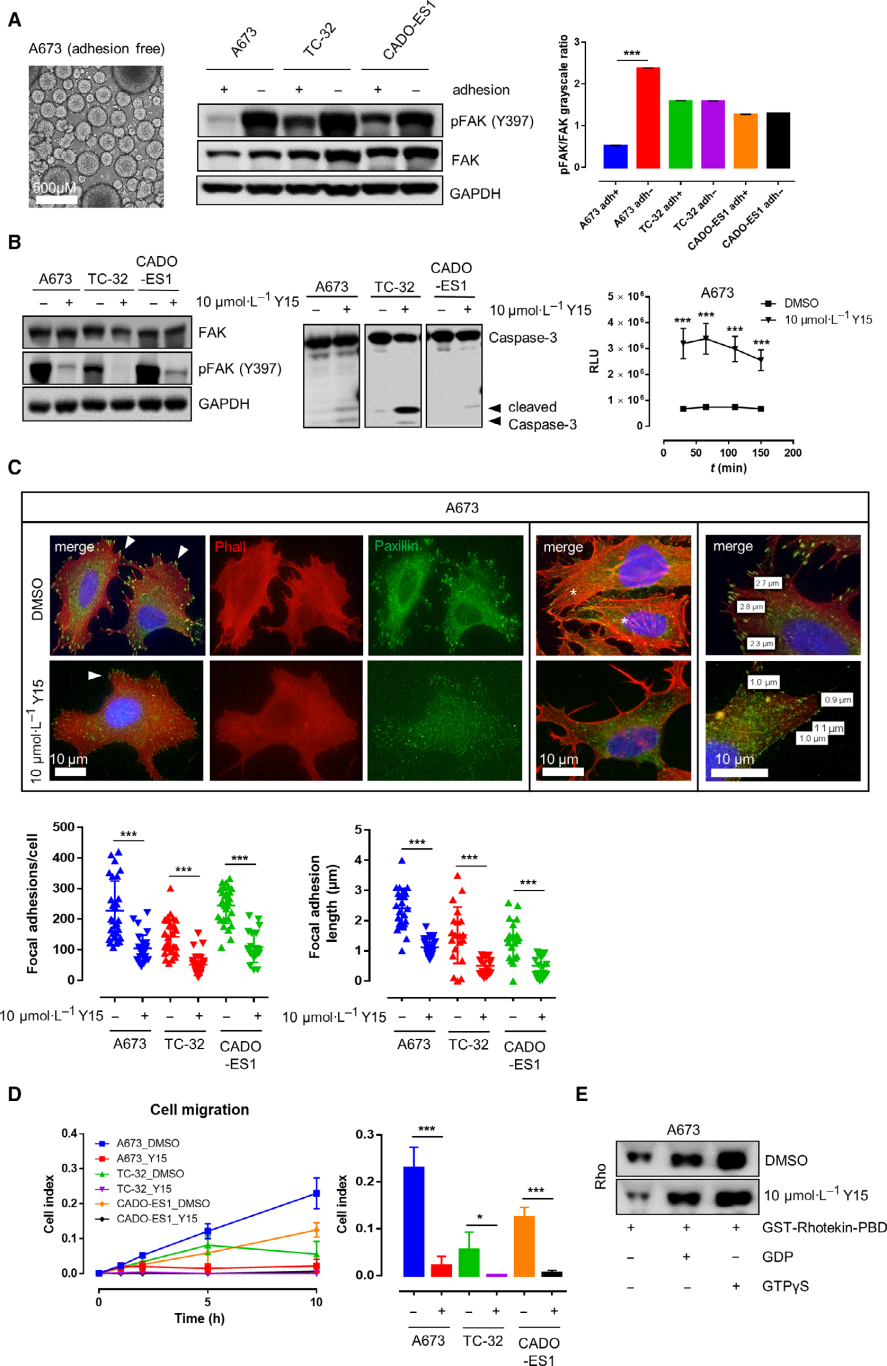
Y397 phosphorylation of FAK under different cell culture conditions and upon application of the FAK inhibitor 1,2,4,5‐benzenetetraamine tetrahydrochloride (Y15). (A) In EwS cell lines grown under adherent and nonadherent conditions, Y397 phosphorylation of FAK was not only persistent, but increased under adhesion‐free conditions in three different EwS cell lines (A673, TC‐32, and CADO‐ES1) as shown by western immunoblotting and densitometric analysis (*n* = 3, Sidak’s multiple comparison test). (B) Application of 10 µm Y15 significantly impaired FAK Y397 phosphorylation and induced cleavage of caspase‐3 in all three EwS cell lines in comparison with DMSO treatment. This was accompanied by a significant increase in apoptosis (ApoTox‐Glo triplex assay, shown here are the results from A673 cells; each *n* = 6, Tukey’s multiple comparison test). (C) imagej software‐based analysis of fluorescence microscopy images showed that treatment of EwS cells with 10 µm Y15 significantly decreased both number (left graph) and size (right graph) of Paxillin‐positive FAs (arrowheads) together with a loss of dorsal stress fibers (asterisks) in all three investigated EwS cell lines (each *n* = 30, Tukey’s multiple comparison test). (D) Cell migration was significantly impaired upon treatment with 10 µm of Y15 compared with DMSO in A673, TC‐32, and CADO‐ES1 cells (each *n* = 6, Tukey’s multiple comparison test). (E) Reduction in migratory capacity of A673 cells upon application of 10 µm Y15 occurred in parallel with a significant decrease in active Rho levels as shown by Rho activity pull‐down assay. *p < 0.05; **p < 0.01; ***p < 0.001.

**Figure 3:**
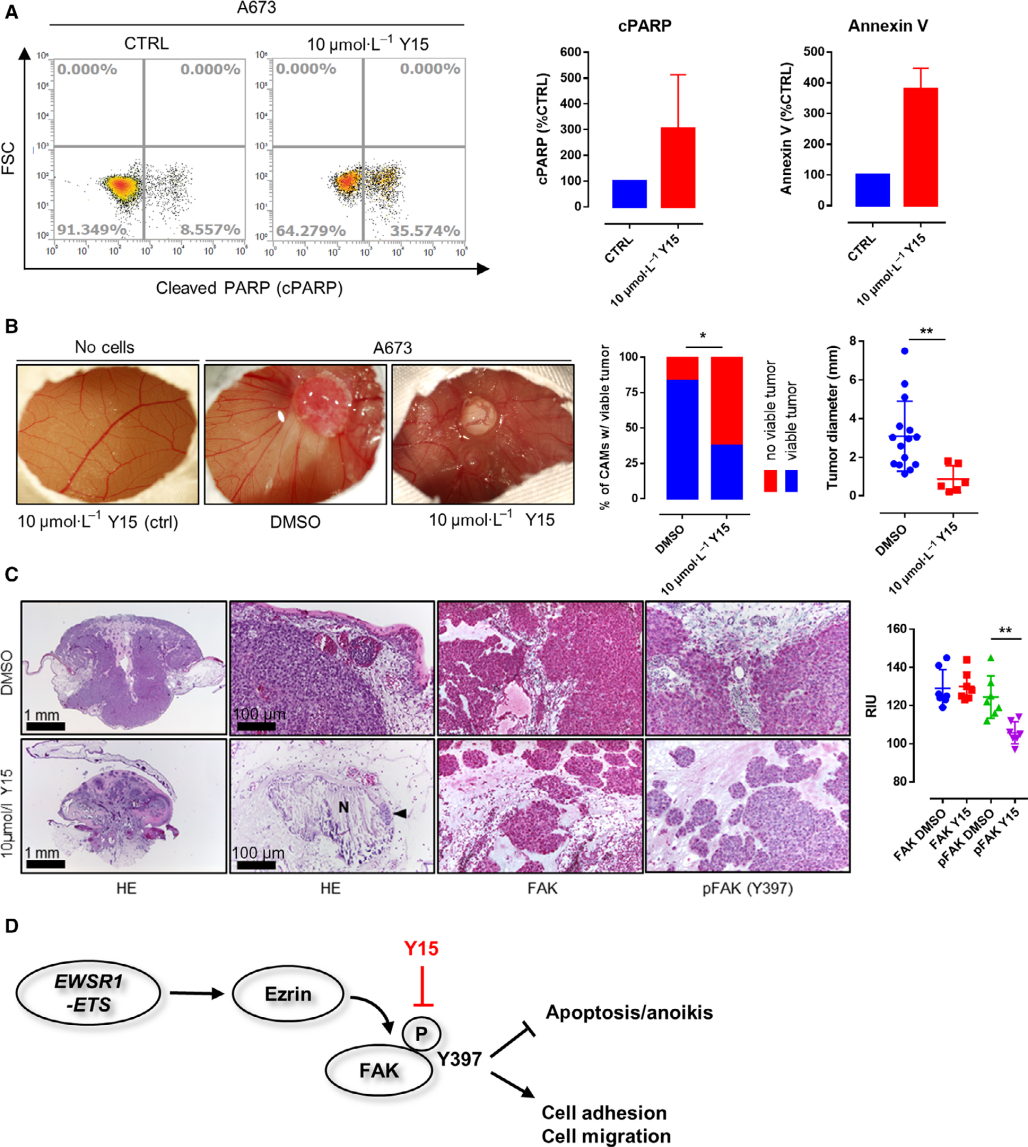
Effects of Y15 treatment on EwS viability and invasion in an *in vivo* (avian CAM) model. (A) FACS analyses confirmed the proapoptotic effect of FAK inhibition on A673 EwS cells. (B) Application of 10 µm Y15 to A673 EwS cells 24h prior to cell seeding significantly impaired the rate of tumor formation as well as tumor size in the CAM model (*P* = 0.0122 and *P* = 0.0095, respectively; *n* = 34 and *n* = 21, Fisher’s exact test and unpaired *t*‐test) while not altering the (micro)vascular architecture of the CAM. (C) Histological analyses showed tumor regression, necrosis (N), and microcalcification with only very few residual tumor cells detectable (arrowhead) upon Y15 treatment. Immunohistochemistry and quantification via reciprocal intensity showed persistent expression of FAK in both Y15‐treated and control cells, but a significant decrease in FAK Y397 phosphorylation in the residual A673 xenograft tumor cells after treatment with Y15 (*n* = 21, Sidak’s multiple comparison test). (D) Schematic diagram of how EWSR1‐FLI1‐dependent expression of Ezrin may contribute to SRC‐independent autophosphorylation of FAK on tyrosine 397 as previously described. The phosphorylation of FAK impairs apoptosis and anoikis and enhances focal adhesion formation and migratory capacity of EwS cell, but can effectively be targeted by application of Y15. *p < 0.05; **p < 0.01; ***p < 0.001.

A673, TC‐32, and CADO‐ES1 remained viable and showed persistent (TC‐32, CADO‐ES1) or enhanced (A673) Y397 phosphorylation of FAK when grown under nonadherent conditions on adhesion‐free hydrogel plates (Fig. 2A), indicating adhesion‐independent FAK phosphorylation in EwS cell lines. IP experiments confirmed interactions between FAK and FA proteins Grb2 and Paxillin in EwS cells *in vitro* (Fig. S2A). Y15, a potent and specific inhibitor of FAK autophosphorylation, effectively abrogated Y397 phosphorylation of FAK in all three EwS cell lines (Fig. 2B). The decrease in FAK phosphorylation upon Y15 treatment occurred in parallel with an increase in caspase‐3 cleavage and apoptosis as shown by immunoblotting and ApoTox‐Glo triplex assays. Along with that, fluorescence microscopy and image analysis revealed a significant decrease in both the number and the size of FAs together with a loss of dorsal actin stress fibers (Fig. 2C). Application of 10 µm Y15 significantly impaired cell migration of A673, TC‐32, and CADO‐ES1 in real‐time cell migration assays using the xCeLLigence system (Fig. 2D) together with a decrease in active Rho levels as shown by Rho activity pull‐down assays (Fig. 2E). The impairment of EwS cell migration upon Y15 application paralleled the effect of FAK siRNA knockdown, underlining the specificity of the employed FAK inhibitor (Fig. S2A,B). There was no effect of FAK inhibition on SRC or other major RTK pathway signaling activity in the investigated cell lines (Fig. S2B). Additional FACS analyses confirmed the proapoptotic effect of FAK inhibition on EwS cells (Fig. 3A).

### 3.5 Y15 impairs viability and invasion of EwS in an *in vivo* (avian CAM) model

A chicken CAM model was used to assess the effects of Y15 on EwS tumor xenografts *in vivo*. Application of 10 µm Y15 alone did not have morphologic effects, particularly with regard to the microvascular architecture of the CAM (Fig. 3B). However, treatment of A673 cells with 10 µm Y15 prior to cell seeding significantly impaired tumor formation (*P* = 0.0122) and led to a significant decrease in the size of invasive experimental EwS (*P* = 0.0095) along with widespread tumor regression, necrosis, and microcalcification with only few residual tumor cell clusters (Fig. 3C). Immunohistochemistry showed the expression of FAK in both Y15‐treated and control cells, but barely detectable Y397 phosphorylation of FAK in the few residual tumor cells after Y15 treatment (*P* < 0.001).

## 4 Discussion

Previous studies suggested a central role for FA reorganization in EwS cell adhesion, migration, and viability (Chaturvedi *et al.*, 2014; Chaturvedi *et al.*, 2012; Wang *et al.*, 2019). In the present study, our aim was to decipher a possible molecular link between EwS‐specific transcription factors (*EWSR1‐FLI1*, E/F) and the expression and/or activation of proteins involved in FA formation and maintenance. Upon E/F knockdown, *Ezrin* mRNA levels were significantly decreased in A673 EwS cells. Ezrin has previously been shown to induce FAK autophosphorylation independent from both SRC signaling and FAK/integrin interaction (Poullet *et al.*, 2001). Correspondingly, xenograft tumors derived from A673 cells after knockdown of E/F with significantly decreased Ezrin mRNA and protein expression showed diminished Y397 phosphorylation of FAK, although overall FAK mRNA and protein expression was unaffected. siRNA‐based knockdown of Ezrin in the EwS cell lines A673, TC‐32 (EWSR1‐FLI1 fusion type 1), and CADO‐ES1 (EWSR1‐ERG fusion gene) (Matsui *et al.*, 2003; Smith *et al.*, 2006) significantly impaired Y397 phosphorylation of FAK in all investigated cell lines. These findings suggest a model in which FAK autophosphorylation in EwS depends on E/F‐driven Ezrin expression, in line with previous results from other authors showing (a) strong expression of Ezrin in primary EwS samples and (b) experimental data supporting a central role of Ezrin in both autophosphorylation of FAK and growth and metastatic spread of EwS (Krishnan *et al.*, 2006; Poullet *et al.*, 2001).

Immunohistochemical analyses of patient samples from the CESS group (*n* = 97) showed moderate‐to‐strong FAK staining in 100% (93/93) and Ezrin staining in 85% (77/90) of evaluable samples, with a high concordance between Ezrin positivity and FAK phosphorylation (*P* < 0.0001, chi‐square test). Surprisingly, there was no significant correlation between FAK expression levels, Ezrin expression, or phosphorylation of FAK and clinicopathological characteristics, such as overall survival, response to chemotherapy, first relapse, or presence of metastatic disease at the time of diagnosis. This lack of clinical association may well be due to the fact that a vast majority of samples in our cohort were Ezrin/pFAK double‐positive (85% and 84%, respectively). However, in concordance with our results, Krishnan *et al.*, investigating a similar percentage of strongly Ezrin‐positive EwS samples in a smaller cohort (20/25, 80%), found no significant differences in the ‘ezrin score’ of patients with localized versus metastatic disease (Krishnan *et al.*, 2006). Thus, the biological impact of Ezrin on the course and outcome of EwS remains to be elucidated as it might be masked by possible alternative signaling pathways in the (small) subgroup of Ezrin‐negative EwS.

Further *in vitro* analyses revealed that A673, TC‐32, and CADO‐ES1 remained viable and showed persistent or even enhanced FAK phosphorylation when grown under nonadherent conditions, which is of special interest since it had previously been shown that Y397 phosphorylation and activation of FAK inhibit anoikis (detachment‐dependent apoptosis) (Frisch *et al.*, 1996). There was no detectable baseline FA phosphorylation on Y576/577 in all investigated EwS cell lines, and baseline Y925 phosphorylation of FAK could only be detected in CADO‐ES1 (Fig. S1F), indicating that Y397 might represent the most relevant FAK tyrosine phosphorylation site in EwS. This is in line with previous reports indicating that the Y397 autophosphorylation site is the primary site responsible for the pro‐migratory, pro‐invasive, and antiapoptotic role of FAK in tumor cells (Megison *et al.*, 2014).

The compounds PF‐573228 (PF‐228) and Y15 are potent and specific inhibitors of FAK autophosphorylation activity (Golubovskaya *et al.*, 2015; Slack‐Davis *et al.*, 2007). Y15 has previously been shown to decrease cell viability and clonogenicity of various carcinomas and to increase detachment, cause apoptosis, and inhibit invasion of glioblastoma cells through the inhibition of FAK phosphorylation (Golubovskaya *et al.*, 2013; Zhang *et al.*, 2016). We could confirm that both Y15 and PF‐228 (data not shown) abrogate Y397 phosphorylation of FAK in EwS; we selected Y15 for further experiments since the *in vivo* activity of the compound had already been documented (Hochwald *et al.*, 2009). Application of 10 µm Y15 effectively impaired FAK phosphorylation on Y397 in all three cell lines. Noteworthy, FAK inhibition had no effect on SRC or other major RTK pathway signaling activity in the investigated cell lines. The decrease in FAK phosphorylation upon Y15 treatment occurred in parallel with an increase in caspase‐3 cleavage and apoptosis (as shown by ApoTox and FACS assay) and a decrease in the number and size of FAs together as well as dorsal actin stress fibers. This decrease in FA formation together with increased caspase‐3‐mediated apoptosis points toward enhanced anoikis in Y15‐treated EwS cells, given that caspase‐3 is one of the key effector molecules of detachment‐dependent cell death (Frisch and Screaton, 2001). Moreover, the application of Y15 significantly impaired cell migration of all investigated EwS cell lines in real‐time cell migration assays together with a decrease in active Rho levels as shown by Rho activity pull‐down assays. This paralleled the results from FAK siRNA knockdown. Since FAK has been identified as a potential therapeutic target in EwS in tyrosine kinase screening assay, and since migration and invasion of EwS depend on the activity of small Rho‐GTPases (Crompton *et al.*, 2013; Krook *et al.*, 2014), these findings support a potential therapeutic role of FAK inhibition in prevention or slowing down metastatic dissemination of EwS.

A chicken CAM model was finally used to assess the effects of Y15 on EwS tumor xenografts *in vivo*. Treatment of A673 cells with 10 µm Y15 prior to cell seeding significantly impaired tumor xenograft formation (*P* = 0.0122) and led to a significant decrease in the size of invasive experimental EwS (*P* = 0.0095) along with loss of FAK phosphorylation, widespread tumor regression, and necrosis.

In summary, the findings presented here lead to a model where *EWSR1‐ETS*‐dependent expression of Ezrin, which can be demonstrated in the vast majority of EwS patient tumor samples, leads to SRC‐independent autophosphorylation of FAK on tyrosine 397 that impairs apoptosis/anoikis and enhances focal adhesion formation as well as Rho‐dependent cell migration of EwS cells. This pathway can be effectively targeted by application of the FAK inhibitor Y15.

## 5 Conclusions

Our results show that key tumorigenic properties of EwS cells depend on Y397 autophosphorylation of FAK, further underlining the crucial role of FA homeostasis in cancer cells (Sulzmaier *et al.*, 2014), and a possible role of FAK inhibitors in the treatment of EwS. The effects of FAK inhibition were independent from the underlying *EWSR1‐ETS* fusion gene. It is well conceivable that autophosphorylation of FAK depends on the structural integrity of the FA protein complex, whose components, such as Ezrin, underlie direct transcriptional control by the E/F oncogene. While further studies should aim at deciphering the exact way how the expression of the oncogenic transcription factor affects FA protein composition in EwS cells, the present work clearly identified FAK autophosphorylation as a potential therapeutic target affecting metastatic dissemination in EwS.

## Conflict of interest

The authors declare no conflict of interest.

## Author contributions

KS, MT, TGPG, and WH conceptualized the data. KS, MT, EPJ, DU, JR, JSG, MFO, and GS curated the data. KS, MT, EPJ, JR, JSG, MFO, GS, MFA, TGPG, and WH were involved in formal data analysis. KS, MT, EW, TGPG, and WH acquired funding. KS, MT, EPJ, JR, JHM, JSG, MFO, GS, MFA, CR, TGPG, and WH investigated the study. JHM, JSG, MFO, GS, MFA, CR, and TGPG contributed to methodology. KS and WH administered the project. JHM, EW, CR, and TGPG provided resources. KS, CR, EW, TGPG, and WH supervised and validated the data. KS wrote the original draft. All authors wrote, reviewed, and edited the manuscript.

## Supporting information


**Fig. S1** **.** Baseline characterization of the EwS cell lines that were used in the study. A, Detection of EWSR1‐FLI1 and EWSR1‐ERG fusion proteins in TC‐32, A673 and CADO‐ES1 cells. B, siRNA‐based knockdown of Ezrin in TC‐32 (48 h) and CADO‐ES1 (48h and 72 h, respectively). C, Compared to TC‐32 and CADO‐ES1, A673 showed the highest proliferative activity and migratory potential in real‐time proliferation and migration assays using the xCeLLigence system. D and E, Software‐based image analysis (imagej) showed numerous Paxillin‐positive FA in the investigated EwS cell lines, with the highest number of FAs observed in A673 and CADO‐ES1. F, FA protein expression in EwS cells. Strong expression and Y397‐phosphorylation of FAK in all investigated EwS cell lines, while FAK Y576/577 expression was only barely detectable. Y925‐phosphorylation of FAK as well as Y118‐phosphorylation of Paxillin were present only in CADO‐ES1 cells.

**Fig. S2** **.** Effect of FAK knockdown on the migratory capacity of EwS cells. A, Effective siRNA‐based knockdown of FAK protein expression in A673 and TC‐32 cells, while there was only a slight decrease of FAK expression in CADO‐ES1 upon siRNA transfection. B, Cell migration was significantly impaired upon FAK siRNA knockdown in A673 and TC‐32 cells, paralleling the effect of FAK inhibition.

**Fig. S3** **.** Protein/Protein interactions of FAK and Src signaling in EwS cells. A, Co‐Immunoprecipitation experiments confirm the protein‐protein interactions between FAK and FA proteins Paxillin and Grb2 in A673 EwS cells. B, FAK inhibition with 10 µm Y15 had no effect on SRC or other major RTK pathway signaling activity in the investigated cell lines (PathScan).

**Table S1** **.** Clinico‐pathological data.

**Table S2** **.** List of antibodies.
