# Supplementary figures and images for: Focal adhesion kinase confers pro‐migratory and antiapoptotic properties and is a potential therapeutic target in Ewing sarcoma
Source: Mol Oncol. 2019 Dec 21;14(2):248–60. doi: 10.1002/1878-0261.12610 (PMC6998388; doi:10.1002/1878-0261.12610)

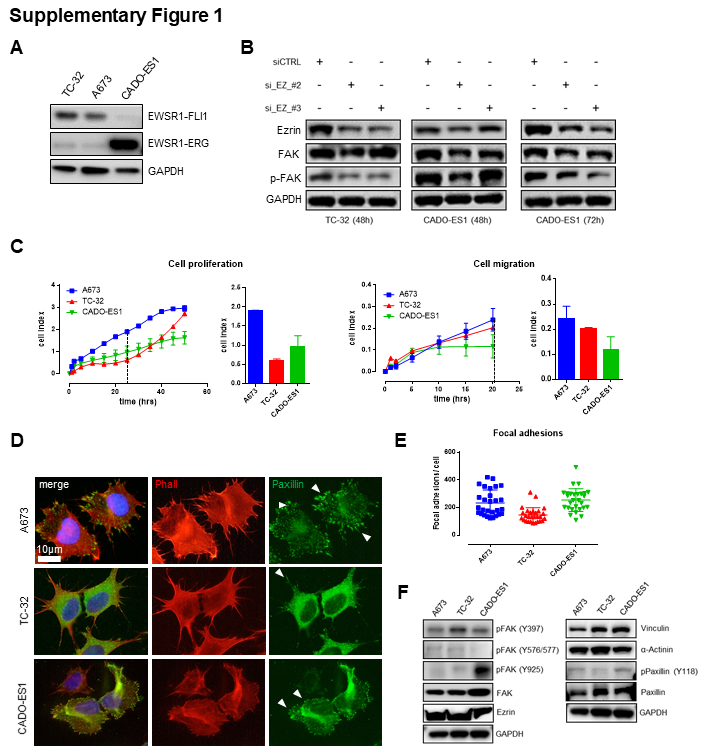

Supplement: Supplementary file 1 — Fig. S1 . Baseline characterization of the EwS cell lines that were used in the study. A, Detection of EWSR1‐FLI1 and EWSR1‐ERG fusion proteins in TC‐32, A673 and CADO‐ES1 cells. B, siRNA‐based knockdown of Ezrin in TC‐32 (48 h) and CADO‐ES1 (48h and 72 h, respectively). C, Compared to TC‐32 and CADO‐ES1, A673 showed the highest proliferative activity and migratory potential in real‐time proliferation and migration assays using the xCeLLigence system. D and E, Software‐based image analysis (imagej) showed numerous Paxillin‐positive FA in the investigated EwS cell lines, with the highest number of FAs observed in A673 and CADO‐ES1. F, FA protein expression in EwS cells. Strong expression and Y397‐phosphorylation of FAK in all investigated EwS cell lines, while FAK Y576/577 expression was only barely detectable. Y925‐phosphorylation of FAK as well as Y118‐phosphorylation of Paxillin were present only in CADO‐ES1 cells. [file MOL2-14-248-s001.tif]

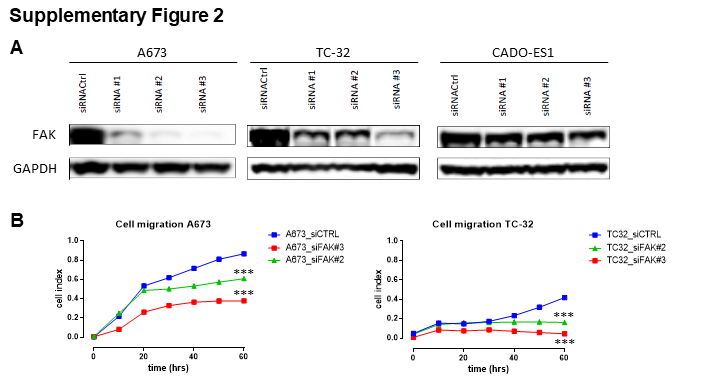

Supplement: Supplementary file 2 — Fig. S2 . Effect of FAK knockdown on the migratory capacity of EwS cells. A, Effective siRNA‐based knockdown of FAK protein expression in A673 and TC‐32 cells, while there was only a slight decrease of FAK expression in CADO‐ES1 upon siRNA transfection. B, Cell migration was significantly impaired upon FAK siRNA knockdown in A673 and TC‐32 cells, paralleling the effect of FAK inhibition. [file MOL2-14-248-s002.tif]

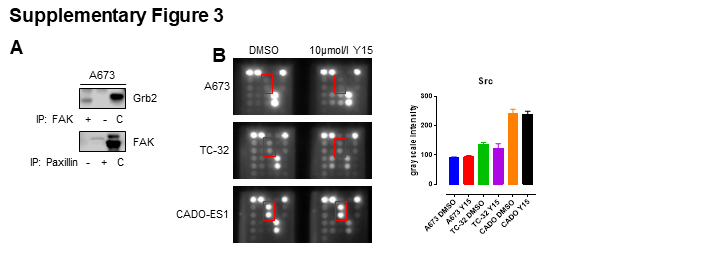

Supplement: Supplementary file 3 — Fig. S3 . Protein/Protein interactions of FAK and Src signaling in EwS cells. A, Co‐Immunoprecipitation experiments confirm the protein‐protein interactions between FAK and FA proteins Paxillin and Grb2 in A673 EwS cells. B, FAK inhibition with 10 µm Y15 had no effect on SRC or other major RTK pathway signaling activity in the investigated cell lines (PathScan). [file MOL2-14-248-s003.tif]
