# Supplementary material for: Focal adhesion kinase confers pro‐migratory and antiapoptotic properties and is a potential therapeutic target in Ewing sarcoma
Source: Mol Oncol. 2019 Dec 21;14(2):248–60. doi: 10.1002/1878-0261.12610 (PMC6998388; doi:10.1002/1878-0261.12610)
Supplement: Supplementary file 5 — Table S2 . List of antibodies. [file MOL2-14-248-s005.docx]

**Supplementary Table 2**

**List of antibodies**

| Antigen | Clone name | Distributor | species | Dil. (WB) | Dil. (IHC) | Dil. (IF) |
| --- | --- | --- | --- | --- | --- | --- |
| α-Actinin | D6F6 | Cell Signaling (Frankfurt, GER) | rabbit monoclonal | 1:1000 |  | 1:100 |
| Caspase-3 |  | Cell Signaling (Frankfurt, GER) | rabbit polyclonal | 1:1000 |  |  |
| Ezrin |  | Cell Signaling (Frankfurt, GER) | rabbit polyclonal | 1:1000 | 1:250 |  |
| FAK | D2R2E | Cell Signaling (Frankfurt, GER) | rabbit monoclonal | 1:1000 | 1:250 |  |
| Phospho-FAK(Tyr397) | D20B1 | Cell Signaling (Frankfurt, GER) | rabbit monoclonal | 1:1000 | 1:250 |  |
| Phospho-FAK(Tyr576/577) |  | Cell Signaling (Frankfurt, GER) | rabbit polyclonal | 1:1000 |  |  |
| Phospho-FAK(Tyr925) |  | Cell Signaling (Frankfurt, GER) | rabbit polyclonal | 1:1000 |  |  |
| FAK | ZF002 | Invitrogen (Rockford, USA) | mouse monoclonal | 1:1000 |  | 1:500 |
| FAK[pY397] | 31H5L17 | Invitrogen (Camarillo, USA) | rabbit monoclonal |  |  | 1:500 |
| Paxillin | D9G12 | Cell Signaling (Frankfurt, GER) | rabbit monoclonal | 1:1000 |  |  |
| Paxillin | 5H11 | Invitrogen (Rockford, USA) | mouse monoclonal | 1:1000 |  | 1:250 |
| GAPDH | D16H11 | Cell Signaling (Frankfurt, GER) | rabbit monoclonal | 1:1000 |  |  |
| Grb2 |  | Cell Signaling (Frankfurt, GER) | rabbit polyclonal | 1:1000 |  |  |
| Phospho-Paxillin (Tyr118) |  | Cell Signaling (Frankfurt, GER) | rabbit polyclonal | 1:1000 |  |  |
